# No evidence of enteric viral involvement in the new neonatal porcine diarrhoea syndrome in Danish pigs

**DOI:** 10.1186/s12917-017-1239-5

**Published:** 2017-11-07

**Authors:** N. B. Goecke, C. K. Hjulsager, H. Kongsted, M. Boye, S. Rasmussen, F. Granberg, T. K. Fischer, S. E. Midgley, L. D. Rasmussen, Ø. Angen, J. P. Nielsen, S. E. Jorsal, L. E. Larsen

**Affiliations:** 1 National Veterinary Institute, Technical University of Denmark, Kemitorvet, Lyngby, DK-2800 Denmark; 2 Department of Biomedical Sciences and Veterinary Public Health (BVF), Swedish University of Agricultural Sciences (SLU), Uppsala, Sweden; 3 Statens Serum Institut (SSI), Artillerivej 5, Copenhagen S, DK-2300 Denmark; 4 Pig Research Centre, Danish Agriculture and Food Council, Vinkelvej 13, DK-8620 Kjellerup, Denmark; 5 National Veterinary Institute, Technical University of Denmark, Lindholm, Kalvehave, DK-4771 Denmark; 6 Department of Veterinary and Animal Sciences, Faculty of Health and Medical Sciences, University of Copenhagen, Gronnegaardsvej 15, DK-1870 Frederiksberg, Denmark

**Keywords:** Diarrhoea, Neonatal piglets, NNPDS, Virus

## Abstract

**Background:**

The aim of this study was to investigate whether the syndrome New Neonatal Porcine Diarrhoea Syndrome (NNPDS) is associated with a viral aetiology. Four well-managed herds experiencing neonatal diarrhoea and suspected to be affected by NNPDS were included in a case-control set up. A total of 989 piglets were clinically examined on a daily basis. Samples from diarrhoeic and non-diarrhoeic piglets at the age of three to seven days were selected for extensive virological examination using specific real time polymerase chain reactions (qPCRs) and general virus detection methods.

**Results:**

A total of 91.7% of the animals tested positive by reverse transcription qPCR (RT-qPCR) for porcine kobuvirus 1 (PKV-1) while 9% and 3% were found to be positive for rotavirus A and porcine teschovirus (PTV), respectively. The overall prevalence of porcine astrovirus (PAstV) was 75% with 69.8% of the PAstV positive pigs infected with PAstV type 3. No animals tested positive for rotavirus C, coronavirus (TGEV, PEDV and PRCV), sapovirus, enterovirus, parechovirus, saffoldvirus, cosavirus, klassevirus or porcine circovirus type 2 (PCV2). Microarray analyses performed on a total of 18 animals were all negative, as were eight animals examined by Transmission Electron Microscopy (TEM). Using Next Generation de novo sequencing (de novo NGS) on pools of samples from case animals within all herds, PKV-1 was detected in four herds and rotavirus A, rotavirus C and PTV were detected in one herd each.

**Conclusions:**

Our detailed analyses of piglets from NNPDS-affected herds demonstrated that viruses did not pose a significant contribution to NNPDS. However, further investigations are needed to investigate if a systemic virus infection plays a role in the pathogenesis of NNPDS.

## Background

Since 2008, field experiences on a new diarrhoeic syndrome in neonatal piglets referred to as New Neonatal Porcine Diarrhoea Syndrome (NNPDS) have been reported in Denmark and elsewhere [1–4]. The prevalence of well-known enteric pathogens as well as gross- and histological findings in age-matched diarrhoeic- and non-diarrhoeic piglets from four Danish herds have recently been reported [5]. In that study, no association between the presence of diarrhoea and the detection of enterotoxigenic *Escherichia coli, Clostridium perfringens* type A or C, rotavirus A, coronavirus, *Clostridium difficile*, *Cryptosporidium spp.*, *Giardia spp.*, *Cystoisospora suis* or *Strongyloides ransomi* was revealed. The conclusion of these detailed examinations was that no known single causative pathogen could be related to the presence of neither clinical disease nor pathological lesions [5, 6].

The aim of the present study was to perform a detailed investigation on possible viral involvement in NNPDS. Selected samples from the previously examined herds were tested for the presence of a range of specific viruses previously found related to enteric conditions in pigs and other species. Viruses that have been shown to cause diarrhoea in pigs are rotavirus, coronavirus, norovirus and sapovirus [7–11], while viruses such as porcine kobuvirus 1 (PKV-1) and the five porcine astrovirus types (PAstV1–5) so far only have been associated with diarrhoea in pigs in a few studies [12–14]. Viruses like enterovirus, parechovirus, saffoldvirus, cosavirus and klassevirus, all belonging to the family *Picornaviridae,* are known human enteric pathogens, but so far there have not been reports on the presence of these viruses in pigs. Porcine teschovirus (PTV) and porcine circovirus type 2 (PCV2) are on the other hand enzootic in pig herds in most countries, but have not been proven to be a primary cause of diarrhoea in pigs [15–18]. Systemic PCV2 may, however, indirectly contribute to enteric diseases due to its immunosuppressive effect [19]. In the present study, samples were examined for the above mentioned viruses by ELISA, conventional reverse transcription PCR (RT-PCR) or real time RT-PCR (RT-qPCR). In addition, selected samples were investigated for the presence of viruses in general by Transmission Electron Microscopy (TEM), pan-viral microarray and de novo sequencing using Next Generation Sequencing (NGS).

## Methods

### Herds and animals

Four well-managed herds affected by severe neonatal diarrhoea for at least one year were selected for the study. Approximately 15 case (diarrhoeic) and 15 control (non-diarrhoeic) piglets per herd were selected for euthanasia. All selected piglets were at the age of three to seven days. In addition, tissue samples from the euthanized animals and a selected number of rectal swabs from the day of euthanasia were included. For details on inclusion criteria and definitions on case and control animals see Kongsted et al. (2013) [5].

### Selection and storing of samples

Live piglets were transported to the laboratory and euthanized within six hours after selection in the herds. Immediately after euthanasia, samples from ileum were snap-frozen on dry ice and stored at −80 °C until further use. Rectal swabs taken in the herds were frozen immediately and kept in a freezer with dry-ice in the herds until transportation to the laboratory, where they were stored at −80 °C until further analyses.

### Nucleic acid extraction

For extraction of RNA for coronavirus (TGEV, PEDV and PRCV) analyses up to 40 mg ileum tissue with content was homogenized in 300 μl chilled 1-Thioglycerol Homogenization Solution (Promega, Nacka, Sweden) in a TissueLyserII (QIAGEN, Copenhagen, Denmark) at 20 Hz for 2 min, vortexed and heated at 70 °C for 2 min and then stored on ice. 300 μl of lysis buffer were mixed into the sample and 10 μl DNase added afterwards. RNA was extracted from all of the homogenate on a Maxwell® automated purification robot with the Maxwell® 16 LEV SimplyRNA Tissue Kit (Promega) according to instructions from the supplier and eluted in 70 μl nuclease free water. The samples were stored at −80 °C until analysis.

Extraction of nucleic acid for PAstV1–5, PKV-1, PTV and PCV2 analyses was performed on samples consisting of ileum with content and on ileum content. For samples of ileum (with content) a 10% homogenate was prepared in RLT plus buffer (QIAGEN). One 5 mm stainless steel bead (QIAGEN) was added to each sample and the samples were homogenized in a TissueLyser II (QIAGEN) for 2 min at 30 Hz. The homogenate was centrifuged for 3 min at 12.000 rpm and the supernatant (400 μL) was used for nucleic acid extraction. For samples of ileum content a 10% homogenate in ATL buffer (QIAGEN) was prepared. One stainless steel bead, 5 mm, was added to each sample and the samples were homogenized in a Tissuelyser II and the supernatant (at least 350 μL) was used for nucleic acid extraction. Extraction of nucleic acid from ileum with content and ileum content samples was automated on the QIAsymphony SP system (QIAGEN) using QIAsymphony RNA kit (QIAGEN) protocol RNA_CT_400_V5 with an elution volume of 100 μL and QIAsymphony DSP virus/pathogen kit (QIAGEN) protocol complex200_V5_DSP without addition of carrier RNA and with an elution volume of 110 μL. The samples were stored at −80 °C until analysis.

Extraction of RNA for rotavirus A and C, norovirus, sapovirus, enterovirus, parechovirus, saffoldvirus, cosavirus and klassevirus was performed on samples consisting of ileum with content. The samples were prepared as a 10% suspension in minimal essential medium and centrifuged at 3500×g for 30 min. Nucleic acids were extracted from 200 μl sample material using the MagNa Pure LC Total Nucleic Acid Isolation Kit (Roche Diagnostics, Hvidovre, Denmark) on the MagNa Pure LC or MagNa Pure 96 (Roche Diagnostics) instruments according to the manufacturer’s specifications. The samples were stored at −80 °C until analysis.

### Detection of viral RNA by RT-PCR or RT-qPCR

In order to detect viral pathogens, samples were tested in different PCR assays. In the RT-qPCR and qPCR assays samples were analysed in duplicates, while samples were analysed as single reactions in the RT-PCR assays. For each separate PCR run positive and negative (nuclease-free water, Amresco) PCR controls were included.

The samples were initially tested by a conventional pan-corona RT-PCR assay designed to detect a wide range of coronaviruses [20]. In house RT-qPCR assays were used to test samples for porcine epidemic diarrhoea virus (PEDV), porcine respiratory corona virus (PRCV) and transmissible gastroenteritis virus (TGEV). TGEV and PRCV were detected simultaneously in a duplex RT-qPCR by combining two published assays [21, 22]. The RT-qPCR was performed as a one-step RT-PCR reaction using the RNA UltraSense ™ One-Step Quantitative RT-PCR System (Invitrogen, Carlsbad, California, USA). Primer and probe concentrations and the PCR assay conditions were as described using the MX3005p qPCR system (Stratagene, Santa Clara, USA). One minor modification was made since the TGEV probe had the fluorescent dye Cy5 at the 5′ end to distinguish its signal from the FAM marked TGEV/PRCV probe. Detection of PEDV was performed under the conditions described by Kim et al.*,* (2007) with the modifications that the RT-qPCR was performed as a one-step RT-PCR reaction using RNA UltraSense ™ One-Step Quantitative RT-PCR System (Invitrogen) and the amplification was performed on the MX3005p qPCR system (Stratagene) [21].

In house RT-qPCR assays were used to test samples for PAstV1–5 and PKV-1 (primer and probe sequences are available from the authors upon request). For detection of PTV, previously published primer (PTV-F: 5CTCCTGACTGGGYAATGGG-3′, PTV-R: 5′-TGTCAGGCAGCACAAGTCCA-3′) and probe (PTV-FAM: 5′-FAM- CACCAGCGTGGAGTTCCTGTAT GGG-BHQ1–3′) sequences were modified and used [23]. PCR amplifications were performed with AgPath-ID™ One-Step RT-PCR Kit (Life Technologies, Carlsbad, California, USA). The RT-qPCR assays were performed in a total volume of 15 μL containing 2 μL template, forward and reverse primer (400 nM each), probe (120 nM), 1× RT-PCR Buffer, 1× RT-PCR Enzyme Mix and nuclease free water. PCR amplification was carried out in RotorGeneQ (QIAGEN) with following thermal cycling conditions: 45 °C for 10 min, 95 °C for 10 min, 45 cycles of 95 °C for 15 s and 60 °C for 45 s.

For each of the RT-qPCR assays specific for PAstV1–5 and PKV-1 a positive standard was used to test the sensitivity of the PCR assays and to quantify the viral load in the samples. The viral load was calculated as log10 to the genome copy number per reaction for the virus.

The samples were furthermore tested for a range of viruses. A previously published RT-qPCR assay targeting the NSP3 gene and designed to detect all rotavirus A genotypes from humans and animals, was used [24]. A published RT-qPCR assay was used to test the samples for rotavirus C virus by targeting the VP7 gene [25]. The presence of norovirus was tested using a previously described multiplex RT-qPCR for Norwalk virus (GI & GII) [26]. The samples were tested for sapovirus by a previously described RT-qPCR assay [27]. Previously described RT-qPCR assays were used to test for enterovirus, parechovirus, saffoldvirus, cosavirus and klassevirus [28, 29].

### Detection of viral DNA by qPCR

Purified DNA was quantified for PCV2 against a standard curve using the Primer Probe Energy Transfer qPCR (PriProEt-RT-PCR) assay as previously described [30].

### ELISA for rotavirus A

Contents of jejunum from all samples were examined for rotavirus group A by an enzyme immunoassay ProSpecT® Rotavirus (Thermo Fisher Scientific, Massachusetts, USA) according to the manufacturer’s instructions.

### Analysis for unknown viruses

For investigation of the samples for the presence of viruses in general, three different methods were used; TEM, pan-viral microarray and de novo NGS.

The TEM analyses were performed on frozen ileum including content from animals with defined and severe villus atrophy previously described by a commercial provider (Bio-imaging unit at Animal and Plant Health Agency (APHA), Weybridge, UK) using standard methods at a magnification of 34,000× [6]. Confirmation of the presence of virus in a sample was based on size, shape, fine structure and surface morphological differentiation.

The microarray analyses were performed on frozen ileum, including content from selected animals with defined and severe villus atrophy as previously described [6]. The microarray consisted of a pan-viral microarray containing 47,000 probes covering all the virus entries in GenBank at the time of its design and was developed in cooperation with APHA in the UK. The protocol for sample preparation and test of samples was as previously described [31]. The estimated sensitivity of the assay was 3–6 log10 copies/reaction.

The de novo NGS analyses were performed on frozen ileum including content from selected animals with defined and severe villus atrophy [6]. The samples were tested in pools of five animals from each of the four herds. Sample preparation and nucleic acid isolation was performed as previously described [32]. By making two parallel extractions of RNA and DNA from each sample, generating cDNA, and pooling the material from all samples in each group, enough material was generated to avoid pre-amplification. Each pooled sample was sequenced on an Ion Torrent PGM system using the 200-bp read chemistry and an Ion 316 chip. This was performed as described earlier [33] at the Uppsala Genome Center, SciLifeLab, Sweden. The resulting reads were assembled using MIRA [34] with the standard settings for de novo assembly of Ion Torrent data. Taxonomic classification of assembled contigs was enabled by Blastn and Blastx searches against local copies of NCBI’s nucleotide and protein databases using the Blast + package [35] with default settings. Evaluating the taxonomic data for potential viruses, candidate reference genomes were identified and retrieved from GenBank in FASTA format. Alignments of contigs against the nucleotide sequences of the reference genomes were performed using the CodonCode Aligner software (CodonCode Corporation).

### Statistical analysis

Logistic regression was used for the comparison of the viral load (copies per reaction) between case and control pigs overall and in the four herds. The outcome in this study was diarrhoea and no diarrhoea. For comparison of the viral load (copies per reaction) between the four herds (herd 1–4), the one-way analysis of variance (ANOVA) test was used. The analyses were performed using R version 3.2.3 [36].

## Results

### Prevalence of enteric viruses in case and control pigs

Samples from a total of 46 case animals and 46 control animals were tested for coronavirus (Table 1). Ileum tissue, ileum contents and rectal swabs from animals in herds 1, 2 and 3 and rectal swabs from animals in herd 4 were tested in the conventional pan-corona assay. All samples tested negative. Some of the samples generated a band at the agarose gel at about the expected size, but subsequent sequencing revealed that the band represented unspecific amplification of porcine DNA (data not shown). Samples from all animals in herds 1–3 (Table 1) were tested in RT-qPCR assay specific for PEDV, PRCV and TGEV with negative results. Ileum samples were omitted from herd 4 in these analyses due to lack of material.

**Table 1:** The number of case and control samples tested in specific and nonspecific virus tests

Herd	Day	Case animals (*n*)	Control animals (*n*)
1	3	7^a^	6
	5	6	7
2	4	2	2
	5	6	6
	7	3	4
3	4	8	7
	6	6	6
4	3	8	6
	5	0	2
Summary		46	46

^a^: One sample was not tested by pan-corona PCR

By ELISA, only one case animal tested positive for rotavirus A. Using RT-qPCR, test of 76 ileum (with content) samples from herds 1–3 (Table 1) revealed a total of seven positive samples, of which six samples were collected from case animals and one from a control animal. The numbers of positive samples in each herd were two, three and two for herds 1, 2 and 3, respectively. The sample that tested positive for rotavirus A in ELISA was the most positive sample in the RT-qPCR assay (quantification cycle (Cq) value of 19). All 76 samples tested for rotavirus C by RT-qPCR yielded negative results. A total of 13 rectal swab samples from case pigs representing all four herds were tested for enterovirus, parechovirus, saffoldvirus, cosavirus, aichivirus and klassevirus by virus specific RT-qPCR assays. All samples yielded negative results. Faecal swab samples from the 76 animals in herds 1–3 (Table 1) were tested in RT-qPCR specific for sapovirus and norovirus with negative results.

### Prevalence of PKV-1, PAstV1–5, PTV and PCV2 in case and control pigs

**Fig. 1:**
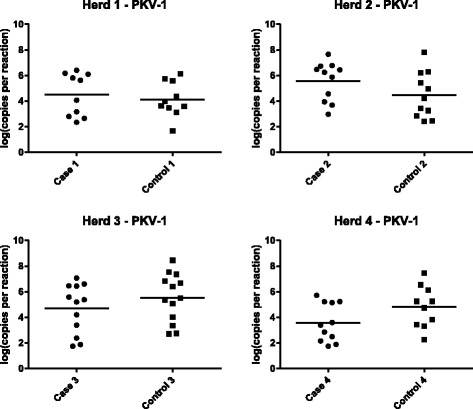
Viral load (log10 copies per reaction) of PKV-1. The copy number for the positive PKV-1 samples in herd 1–4 for case and control pigs. The straight line shows the mean value

Ileum samples from a total of 47 case pigs and 49 control pigs were tested for PKV-1 in a specific RT-qPCR assay. The overall prevalence of PKV-1 was found to be 91.7% (88/96) and the virus was detected in all four herds with prevalence ranging between 80 and 100%. The overall prevalence for the case pigs was 93.6% compared to 89.8% for the control pigs (Table 2). PKV-1 had a similar mean value (±SD) of viral load for the case (4.60 ± 1.76) and control (4.79 ± 1.72) pigs (Table 3). The log-transformed copy numbers for the positive samples are shown in Fig. 1 for the four herds. No statistically difference in viral load between the case and control pigs was observed for PKV-1 (*p* > 0.05) overall or in the four herds (Table 3).

**Table 2:** Prevalence of PKV-1 and PAstV1–5. The number of positive samples, followed by percentage in parentheses

Herd	Status	No. of samples	PKV-1	PAstV1	PastV2	PastV3	PastV4	PAstV5
1	Case	12	10/12 (83.3%)	0/12 (0%)	0/12 (0%)	5/12 (41.7%)	2/12 (16.7%)	1/12 (8.3%)
	Control	13	10/13 (76.9%)	0/13 (0%)	0/13 (0%)	13/13 (100%)	0/13 (0%)	0/13 (0%)
2	Case	11	11/11 (100%)	0/11 (0%)	0/11 (0%)	11/11 (100%)	4/11 (36.4%)	2/11 (18.2%)
	Control	11	11/11 (100%)	0/11 (0%)	0/11 (0%)	11/11 (100%)	1/11 (9.1%)	4/11 (36.4%)
3	Case	12	12/12 (100%)	0/12 (0%)	0/12 (0%)	7/12 (58.3%)	3/12 (25%)	3/12 (25%)
	Control	13	13/13 (100%)	0/13 (0%)	0/13 (0%)	6/13 (46.2%)	1/13 (7.7%)	2/13 (15.4%)
4	Case	12	11/12 (91.7%)	0/12 (0%)	0/12 (0%)	7/12 (58.3%)	0/12 (0%)	0/12 (0%)
	Control	12	10/12 (83.3%)	0/12 (0%)	0/12 (0%)	7/12 (58.3%)	0/12 (0%)	0/12 (0%)
Summary	Case	47	44/47 (93.6%)	0/47 (0%)	0/47 (0%)	30/47 (63.8%)	9/47 (19.2%)	6/47 (12.8%)
	Control	49	44/49 (89.8%)	0/49 (0%)	0/49 (0%)	37/49 (75.5%)	2/49 (4.1%)	6/49 (12.2%)
Total		96	88/96 (91.7%)	0/96 (0%)	0/96 (0%)	67/96 (69.8%)	11/96 (11.5%)	12/96 (12.5%)

**Table 3:** Viral load (log_10_ copies per reaction) of PKV-1 and PAstV3–5. The values represent Mean ± SD

Herd	Status	PKV-1	PAstV3	PAstV4	PAstV5
1	Case	4.52 ± 1.66	6.45 ± 1.20	5.09 ± 0.01	5.54
	Control	4.13 ± 1.36	8.36 ± 2.23	–	–
2	Case	5.58 ± 1.52	9.07 ± 1.04^a^	5.72 ± 0.52	4.53 ± 0.32
	Control	4.49 ± 1.79	7.15 ± 2.40^a^	4.82	5.05 ± 0.40
3	Case	4.70 ± 1.93	6.77 ± 1.35	5.83 ± 1.03	5.29 ± 0.63
	Control	5.54 ± 1.88	7.85 ± 1.38	5.39	4.93 ± 0.01
4	Case	3.59 ± 1.50	6.52 ± 1.90	–	–
	Control	4.82 ± 1.63	8.18 ± 2.21	–	–
Summery	Case	4.60 ± 1.76	7.50 ± 1.78	5.62 ± 0.68	5.08 ± 0.61
	Control	4.79 ± 1.72	7.89 ± 2.15	5.11 ± 0.40	5.01 ± 0.32

^a^ A statistical significant difference (*p* = 0.032) between the viral load for the case and control pigs

Ileum samples from a total of 47 case pigs and 49 control pigs were tested in five RT-qPCR assays specific for each of the five PAstV types. In total, 75% (72/96) of the animals were positive for at least one of the PAstVs. Among the PAstV types, PAstV3 had the highest overall prevalence of 69.8%, followed by PAstV5 (12.5%) and PAstV4 (11.5%) while none of the samples tested positive for PAstV1 or PAstV2. PAstV3 was detected in all four herds, while PAstV4 and PAstV5 only were found in herds 1–3 (Table 2).

The overall prevalence of PAstVs in the case pigs was 70.2% compared to 79.6% for the control pigs. PAstV3 was found in 63.8% of the case pigs and in 75.5% of the control pigs. For PAstV4 and PAstV5, 19.2% and 12.8% of the case pigs were positive, respectively. In comparison, 4.1% and 12.2% of the control pigs were positive for these two viruses (Table 2).

**Fig. 2:**
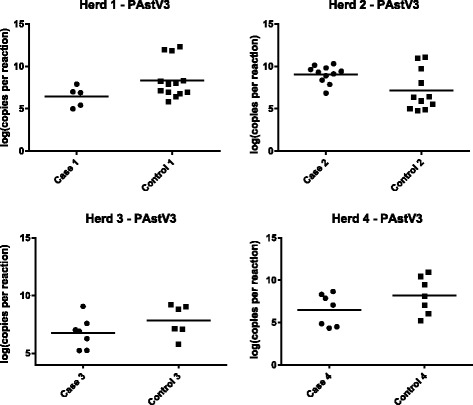
Viral load (log10 copies per reaction) of PAstV3. The copy number for the positive PAstV3 samples in herd 1–4 for case and control pigs. The straight line shows the mean value

The mean value (±SD) of viral load for PAstV3 was 7.50 ± 1.78 for the case pigs and 7.89 ± 2.15 for the control pigs (Table 3). The log-transformed copy numbers for the positive samples are shown in Fig. 2. No statistically difference was observed between the viral load in the case and control pigs for PAstV3 (*p* > 0.05) overall. However, examining of the individual herds using logistic regression showed that there was a statistical significant difference between the viral load for PAstV3 for the positive case and control pigs in herd 2 (*p* = 0.032), while no significant difference was observed in herd 1 (*p* = 0.17), 3 (*p* = 0.87), or 4 (*p* = 0.59). The mean value (±SD) of viral load for PAstV4 was 5.62 ± 0.68 and 5.11 ± 0.40 for the case and control pigs (Table 3), respectively. For PAstV5 the values were 5.08 ± 0.61 and 5.01 ± 0.32 for the case and control pigs (Table 3). No statistically difference was observed between the viral load in the case and control pigs for either PAstV4 or PAstV5.

Furthermore, the 47 case and 49 control samples were tested for PTV and PCV2 with a specific RT-qPCR and qPCR assay, respectively. Three out of 96 animals tested positive for PTV, whereas none of the pigs tested positive for PCV2 (data not shown).

### Co-infection with different viruses

Viral co-infection with two or more viruses was observed in all herds. Different combinations of PAstVs were detected in 15 of the 96 pigs (15.6%) (Table 4), while co-infection with other viruses was found in seven case pigs and one control pig. These pigs were infected with different combinations of rotavirus A, PTV, PKV-1 and PAstVs.

**Table 4:** Co-infection with two or three PAstV types in the same pig

Herd	No. of samples	PAstV3 + V4	PAstV3 + V5	PAstV4 + V5	PAstV3 + V4 + V5
1	25	1/25 (4%)	1/25 (4%)	0/25 (0%)	0/25 (0%)
2	22	3/22 (13.6%)	4/22 (18.2%)	0/22 (0%)	2/22 (9.1%)
3	25	2/25 (8%)	0/25 (0%)	1/25 (4%)	1/25 (4%)
4	24	0/24 (0%)	0/24 (0%)	0/24 (0%)	0/24 (0%)
Total	96	6/96 (6.3%)	5/96 (5.2%)	1/96 (1.0%)	3/96 (3.1%)

### Nonspecific virus tests

For investigation of the presence of virus in general, selected samples were analysed with TEM, pan-viral microarray and de novo NGS. Eight samples from case animals with histological lesions typical of viral infections (villus atrophy) representing all four herds were analysed by TEM with negative results. A total of 18 samples from 13 case and five control animals were tested by microarray. None of the samples tested conclusively positive for any of the viruses present on the pan-viral microarray (data not shown). Samples from five case animals from each herd were pooled and tested by de novo NGS. Endogenous retrovirus was present in all four pools (data not shown). PKV-1 was also present in all four pools as evident by several reads (Table 5). In addition, the pools of samples from herd 1 were positive for rotavirus C, herd 2 were positive for rotavirus A and herd 3 were positive for PTV. One of the samples included in the pool from herd 2 was from the animal that also tested positive for rotavirus A by RT-qPCR and by ELISA.

**Table 5:** Results of Next Generation Sequencing

Herd	Case animals (*n*)	Result of test of pools of 5 samples
1	5	Porcine rotavirus (group C) - [1 Contig, 26 reads]
		Porcine kobuvirus - [6 Contigs, 206 reads]
2	5	Rotavirus (group A) - [1 Contig, 10 reads]
		Porcine kobuvirus - [1 Contig, 1282 reads]
3	5	Porcine teschovirus - [3 Contigs, 288 reads]
		Porcine kobuvirus - [16 Contigs, 1479 reads]
4	5	Porcine kobuvirus - [11 Contigs, 262 reads]

## Discussion

The present study is part of a series of studies focusing on identifying the cause of NNPDS in Danish pigs, which has previously been shown not to be clearly associated with known bacterial or parasitic pathogens [5, 6]. In the present study the tests for viruses were broadened by testing selected samples for a range of specific viruses linked to enteric disorders in pigs or humans.

The initial test of the samples for rotavirus A virus by a commercial available ELISA generated only one positive sample [5]. Totally seven of 76 (9.2%) samples tested positive when retested in a RT-qPCR assay specific for rotavirus A. The difference between the outcome of tests by ELISA and RT-qPCR most likely reflects the higher sensitivity of the PCR, which was also underlined by the fact that the ELISA positive sample was the sample with the lowest Cq value in the PCR. The low number of positive samples strongly indicated that rotavirus A was not a significant problem in very young animals in the NNPDS herds of the present study. None of the samples from pigs younger than one week of age were positive for rotavirus C by RT-qPCR, but one pool of samples, from the pigs in herd 1, investigated by de novo NGS generated 26 reads matching rotavirus C. The differences in the outcome of the two tests can be due to the specificity of the primers and probe used in the PCR. Since the assay was designed based on sequences specific for human rotavirus C it is unclear if the negative results in the PCR test reflected a low level of rotavirus C among Danish pigs or if the assay used did not detect porcine rotavirus C. Group C rotavirus was first identified in swine in 1980 [9], and diarrheal outbreaks associated with rotavirus C have been documented in nursing, weaning and post-weaning pigs, either alone or in mixed infection with other enteric pathogens [37]. In a recent study in the USA, 19.5% of the tested samples were found positive with a higher rotavirus C frequency in case (28.4%) piglets compared to non-case (6.6%) piglets [38]. In contrast, the combined results of the RT-qPCR and the NGS of the present study indicated that this virus did not contribute to NNPDS.

Testing of samples for the two porcine coronaviruses TGEV and PEDV generated the expected negative results. Denmark is considered free of these viruses, but PEDV has recently re-emerged as a significant pathogen in Asia and North America [39] and has also been detected in several European countries including Germany [40].

The testing of samples for sapovirus gave negative results, indicating that sapovirus did not contribute to NNPDS. Porcine sapovirus has been shown experimentally to induce mild to moderate diarrhoea in pigs [41, 42], but epidemiological evidence for a causative role of sapovirus in natural cases of suckling pig diarrhoea is scarce. In a European survey from 2010, sapovirus was detected in faecal samples in 1.6%, 12.2% and 43.4% of suckling pigs from Hungary, Spain and Slovenia, respectively, but there was no association between diarrhoea and detection of sapovirus [43]. In the same study, faecal samples from 57 two-eight weeks old pigs with diarrhoea from 31 Danish herds were tested for sapovirus, with positive results in 68% of the herds, covering 44% of the pigs. The test used both in the European study and the present Danish study was identical [27], however, the pigs included in the present study were younger than the pigs tested in the previous study, which may explain the difference in test results.

Norovirus was not detected in any of the samples. Norovirus has been shown to be prevalent in slaughter pigs in USA [44] and in Belgium [45] whereas samples from pigs aged 1–16 weeks tested negative in Spain [46]. In 2007, a screening of 56 routine laboratory submissions from 31 Danish swine herds revealed that 16% of the herds were positive (unpublished results). The age of the pigs included in the screening was not known, but the combined results indicate that porcine norovirus is indeed prevalent in Danish herds, and the negative outcome of the test described in the present study is probably due to the young age of the animals tested.

Thirteen of the samples from case pigs were screened in a multiplex qPCR, developed for diagnostic use in humans, for the suspected emerging human enteric pathogens enterovirus, parechovirus, saffoldvirus, cosavirus and klassevirus. These samples were negative, and since there have been no reports on these virus being present in pigs it was decided to omit testing of the remaining samples.

The viruses PAstVs and PKV-1 were found with high prevalence in the present study using RT-qPCRs. These viruses have been detected both in diarrhoeic and healthy pigs worldwide, and their role as causative agents of diarrhoea in pigs is not established [10, 47–52], however, some studies have found an association between detection of either PAstVs or PKV-1 and diarrhoea in pigs [12–14]. In the present study, a high overall prevalence of PAstVs was detected, with PAstV3 as the most prevalent type in all four herds. PAstV4 and PAstV5 were present in three herds, while PAstV1 and PAstV2 were not detected in any of the herds. No statistical significant difference between the viral load in the case pigs and control pigs was observed when looking at the data overall. So based on this analysis, there was no evidence for the case pigs having a higher viral load than the control pigs or that detection of PAstV led to diarrhoea in the pigs. In herd 2, however, there was a significant higher viral load of PAstV3 in the case pigs compared to the control pigs. In this herd all pigs were found to be positive for PAstV3, so in this herd it was not the presence of the virus but merely the amount of virus that could be related to diarrhoea. Nevertheless, the results did not support that PAstVs are generally involved in NNPDS.

The presence of two or three PAstV types in the same pig was found in a few of the samples, which is in accordance with previous findings [49]. An overall high prevalence of PAstV in pigs has been observed elsewhere, but to our knowledge this study is the first to detect PAstV3 as the dominant type. PAstV3 has been found in USA, Canada, Croatia and East Africa, but only in few of the tested pigs [47, 49, 53–55] and some of these studies found PAstV3 to be most prevalent in young piglets. Xiao and co-workers found the highest prevalence (4.72–5.3%) of PAstV3 in suckling pigs (0–20 days), and in the study by Luo et al.*,* 2011, PAstV3 was only detected in this age group [47, 49]. Thus, the results in this study also suggested an association between PAstV3 and the early growing stage of the pigs. However, further investigations are needed to confirm if this is the case in all herds. A high overall prevalence of PAstVs has been detected in Croatia (89%), Canada (79.2%), USA (64%), Czech Republic (34.2%) and Germany (20.8%), where the most prevalent PAstV type differs between the countries [47, 49, 54, 56, 57].

In the present study, 91.7% of the animals tested positive for PKV-1 by RT-qPCR. The virus was found in all four herds, but no statistical significant difference between the viral load in the case and control pigs was observed either overall or at herd level. The PCR result was consistent with the findings by the de novo NGS, where PKV-1 also was detected in all herds. A high prevalence in young piglets has also been observed by others, and it has been indicated that there is a higher rate of infection with PKV-1 in young pigs than in older pigs [50, 51, 55]. A study conducted in 40 Korean herds detected a higher prevalence of PKV-1 in diarrhoeic vs. non-diarrhoeic piglets [13]. However, only 4% of the diarrhoeic pigs were not co-infected with other pathogens. Recent studies have found the prevalence of PKV-1 to be similar in diarrhoeic and healthy pigs [58–61], although, two of the studies showed that diarrheic suckling piglets was the most frequently infected group but the results were not statistically significantly different from the healthy piglets [58, 59]. Altogether, these finding indicated that PKV-1 is not a significant primary pathogen in natural cases of diarrhoea in young piglets.

Only few samples were positive for PTV. This result is consistent with the de novo NGS, where only case samples were included, which also found herd 3 to be PTV positive. To date, 13 serotypes of PTV have been associated with a variety of clinical diseases. PTV-1 strains were associated with highly fatal, non-suppurative encephalomyelitis of pigs (Teschen disease) in the 1930–1950s. Today, less virulent talfan strains of PTV-1 are more widespread, and PTVs are detected in swine herds worldwide often together with a variety of other common swine pathogens. In a Chinese study, PTV was found in 96.7% of 30 culled four-eight weeks old post-weanling piglets by nested RT-PCR, but this study concluded that PTV was only marginally related to non-suppurative encephalitis [62]. However, other PTV types may cause other disease symptoms [63].

None of the samples were positive for PCV2. This virus is regarding the primary causative agent of postweaning multisystemic wasting syndrome (PMWS), which has had a huge influence on the pig production worldwide during the last 10 years [64, 65]. So far, PCV2 has not been shown to be a primary causative agent of diarrhoea, and the clinical description of PMWS related diarrhoea is considered to be a result of the lymphoid depletion, which is seen during PCV2 infections. This can lead to immunosuppression which may predispose for co-infections with other pathogens leading to diarrhoea [18, 66]. PCV2 related diseases have not been described in very young piglets so the negative findings in the present study were not surprising.

During recent years, newer techniques using a metagenomic approach, such as microarrays and de novo NGS, have been developed and used to detect emerging and re-emerging viruses in humans and in animals [33, 67]. The advantages of these techniques are that they can detect all viruses irrespective of prior knowledge of the virus genetic sequence. The only prerequisite is that the new virus has some level of identity to previously sequenced viruses in order to be picked up by the downstream bioinformatic filtering of the data [68]. The general virological analyses employed in the present study were so expensive that it was not economically possible to test all samples. The pan viral microarray chip used in the present study included more than 40.000 probes covering all the viruses present in the GenBank [31]. The array failed to detect any virus in the relatively few (*n* = 18) samples tested, indicating that no, or small amounts of, virus particles were present. The detection level of the chip is low compared to qPCR and de novo NGS [69]. In accordance, eight of eight samples examined by TEM, which also has a relatively poor level of detection, also gave negative results.

Compared to other studies on pig faeces, very few viruses were detected by de novo NGS in the present study. A previous study conducted on a US farm detected PKV-1 (23% of all reads), PAstV (22%), enterovirus (14%), sapoviruses (5.7%), sapeloviruses (1.5%), coronaviruses (0.69%), bocaviruses (0.22%) and PTV in 0.03% of the reads [70]. In the present study only PKV-1, PAstV and PTV were detected. Interestingly, the US study found that, except for PKV-1, the prevalence of most viruses was not greater in animals with diarrhoea than in control animals. The difference in viral prevalence between the studies may be explained by difference in age of the animals tested, but the most likely explanation is that the US study used random PCR amplification prior to library building, which greatly improves the sensitivity but also introduces a bias, which can prevent a meaningful quantitative analysis [71]. The sensitivity of the NGS protocol used for each of the pathogens has not been determined so it is possible that a low copy virus has been missed. Experimental infections of animals with filtered faeces from affected animals would be a way to investigate if viral components contribute to the NNPD syndrome, but the pilot studies performed by the project group gave inconclusive results (unpublished observations).

## Conclusion

In conclusion, the analyses performed on samples from four Danish pig herds in this study did not find a significant contribution of enteric viruses to NNPDS. Despite the fact that a high prevalence and viral load of PAstV3 and PKV-1 were detected, these viruses did not seem to be the causative agents of NNPDS. However, since only enteric samples were analysed, further studies are needed to investigate whether a systemic virus infection may play a role in the pathogenesis of NNPDS.
